# The Effect of SEBS/Halloysite Masterbatch Obtained in Different Extrusion Conditions on the Properties of Hybrid Polypropylene/Glass Fiber Composites for Auto Parts

**DOI:** 10.3390/polym13203560

**Published:** 2021-10-15

**Authors:** Zina Vuluga, Catalina-Gabriela Sanporean, Denis Mihaela Panaitescu, George Mihail Teodorescu, Mihai Cosmin Corobea, Cristian Andi Nicolae, Augusta Raluca Gabor, Valentin Raditoiu

**Affiliations:** 1 National Institute for Research and Development in Chemistry and Petrochemistry-ICECHIM, Polymer Department, 202 Spl. Independentei, 060021 Bucharest, Romania; george.teodorescu@icechim.ro (G.M.T.); mihai.corobea@icechim.ro (M.C.C.); cristian.nicolae@icechim.ro (C.A.N.); raluca.gabor@icechim.ro (A.R.G.); vraditoiu@icechim.ro (V.R.); 2 Department of Materials and Production, Aalborg University, Fibigerstraede 16, DK-9220 Aalborg East, Denmark; gabi.potarniche@gmail.com

**Keywords:** polypropylene, SEBS, hybrid composites, mechanical properties, nanoindentation, nanoscratch, glass fiber

## Abstract

Masterbatches from a linear poly[styrene-b-(ethylene-co-butylene)-b-styrene] (SEBS) and halloysite nanotubes (HNT-QM) were obtained in different conditions of temperature and shear using two co-rotating twin-screw extruders. The influence of screw configuration and melt processing conditions on the morpho-structural, thermal and mechanical properties of masterbatches at macro and nanoscale was studied. A good dispersion of halloysite nanotubes and better thermal stability and tensile and nanomechanical properties were obtained at a lower temperature profile and higher screw speed. The effect of masterbatches, the best and worst alternatives, on the properties of a polypropylene (PP)–glass fiber (GF) composite was also evaluated. Double hardness, tensile strength and modulus and four times higher impact strength were obtained for PP/GF composites containing masterbatches compared to pristine PP. However, the masterbatch with the best properties led further to enhanced mechanical properties of the PP/GF composite. A clear difference between the effects of the two masterbatches was obtained by nanoindentation and nanoscratch tests. These analyses proved to be useful for the design of polymer composites for automotive parts, such as bumpers or door panels. This study demonstrated that setting-up the correct processing conditions is very important to obtain the desired properties for automotive applications.

## 1. Introduction

Polypropylene (PP) is a low-cost thermoplastic polymer with wide application in the packaging, automotive, electronics and construction industries. The forecasted market size of polypropylene is USD 99 billion for 2022 [1]. PP nanocomposites with different inorganic fillers have been developed to improve the properties of neat PP and to enlarge its application field [2,3]. Reaching the best properties in PP nanocomposites with montmorillonite (MMT), halloysite nanotubes (HNT) or kaolinite is still a challenge due to the poor dispersion of nanofillers and week adhesion between the hydrophobic PP and high polarity clays [4]. Strategies to overcome this problem have been reported including grafting hydrophilic groups to the polymer matrix, surface modification of nanofillers or coupling agents’ addition [2,3,4,5,6]. Thus, PP nanocomposites were obtained using MMT, HNT or sepiolite grafted with two organosilanes, 3-aminopropyltriethoxysilane (APTES) and vinyltrimethoxysilane [4]. Different properties were noticed depending on the treatment and clay type, a consistent improvement of the Young’s modulus being observed only with APTES modified clays [4]. In the case of kaolinite, which was a surface modified with two types of silane coupling agents, n-octyltriethoxysilane and isobutyltrimethoxysilane, only the second treatment led to PP-kaolinite nanocomposites with improved Young’s modulus and flexibility [6].

Thermoplastic elastomers such as styrene block copolymers are special modifiers, which may act as compatibilizers and impact modifiers in PP, also improving the dispersion of nanofillers [7,8,9,10]. A good dispersion of nanosilica in the interlamellar regions of the PP matrix was observed in melt processed PP/poly(styrene-b-ethylene butylene-b-styrene) (SEBS)/nanosilica nanocomposites containing malleated polypropylene (MAPP) as a coupling agent [7]. Similarly, it was demonstrated that organically modified MMT was located in the SEBS domains in PP/SEBS/clay nanocomposites [8]. A synergistic effect of SEBS (23%) and nanoclay was observed in PP/SEBS/nanoclay nanocomposites, which showed a significant increase in elongation at break and impact strength [9]. By varying the concentration of SEBS and nanoclay in a wide range, nanocomposites with different toughness–stiffness properties, interesting for several industrial applications, were obtained [10].

The automotive industry requires high performance lightweight materials characterized by a high level of mechanical properties for replacing classical metallic and non-metallic parts. This combination of properties can be achieved only at high glass fibers (GF) loadings [11]. PP reinforced with 20–50% GF was intensively studied for different applications [11,12]. A synergistic effect was observed in PP hybrid composites containing both GF and nanoparticles [13,14,15]. Thus, a higher stiffness was noticed for PP/(treated or untreated) MMT/GF hybrid composites compared to the composites without clay [13,14]. Similar improvement was reported for hybrid composites based on PP, GF and HNT in the presence of MAPP as a compatibilizer: the addition of 2.5% HNT increased the tensile strength and modulus of PP/20%GF composites, especially in the presence of MAPP [15].

For some car parts, such as bumpers or side-doors, a high toughness is required in addition to a good strength and stiffness. For this purpose, SEBS was added in PP/GF composites, determining a significant increase of the impact strength at the expense of strength and stiffness [16,17]. Thus, an increase of the impact strength with more than 200% was noticed when 15% maleic anhydride grafted SEBS (SEBS-MA) was added in PP/20% GF composite [17]. It was similar for PP/30%GF composites modified with 20% SEBS or SEBS-MA [16]. However, the tensile strength decreased after SEBS addition, and the elastic modulus was diminished regardless the elastomeric modifier, SEBS or SEBS-MA [16].

Although the effect of SEBS on the properties of PP/GF composites was reported in several works [16,17] and the effect of nanoclay addition on the properties of SEBS was also studied [18,19], no information on the simultaneous effect of GF, HNT and SEBS on PP properties was reported so far. Moreover, no study on the influence of the manufacturing process, equipment and molding conditions on the properties of PP/SEBS/HNT/GF hybrid composites was ever done, although tuning these parameters is essential to obtain materials with optimum properties for auto parts.

Melt processing by extrusion is the preferred industrial route to obtain polymer (nano)composites owing to eco-friendliness, lack of toxic or expensive solvents and cost efficiency in the manufacture of large amounts [20,21]. A higher residence time and adequate shear intensity are necessary for the intercalation or exfoliation of nanoclays in PP nanocomposites [21,22] but a combination of high shear rate and long residence time may have a contrary effect [23]. Indeed, the screw profile and the extrusion conditions have a strong influence on the dispersion of nanoparticles in the polymer matrix [24]. In particular, exfoliation could be achieved even at low shear rate if temperature is high enough, facilitating the diffusion of the polymer into clay galleries. Accordingly, the critical factors which influence the processing of nanocomposites are the screw configuration, selection of temperature profile and screw rotating speed [23].

The advantage of using a masterbatch instead of adding nanoparticles directly in PP is well documented [15,20,21,22,23]. However, all these masterbatches are based on the PP used as polymer matrix [15,20], a mixture of PP with MAPP [22] or only MAPP [21,23]. SEBS was scarcely studied to obtain HNT masterbatches intended for PP nanocomposite fabrication. In one attempt, a SEBS-MA/HNT masterbatch was prepared by solution casting using tetrahydrofuran as a solvent, and then, it was diluted with PP using a melt mixer [25,26]. Unlike direct mixing method, the masterbatch route resulted in a good dispersion of HNT in PP, higher impact strength but lower tensile strength [25,26]. In another attempt, PP/SEBS/MMT nanocomposites were obtained with a twin-screw extruder using different addition protocols; mixing first the MMT with SEBS and then with PP led to the highest impact strength and lowest tensile strength [27].

However, the influence of the processing conditions on the properties of SEBS/HNT masterbatches was never investigated. Although a high shear rate during melt processing is important for a good dispersion of nanoparticles, this may also lead to a significant degradation of SEBS [28]. Setting-up the correct processing conditions to obtain the desired properties of nanocomposites is very important in automotive application. Therefore, the role of melt processing conditions, i.e., screw rotation speed and temperature profile together with the screw configuration on the structure and properties of SEBS/modified halloysite nanotubes (HNT-QM) masterbatches was studied in this work. In addition, the influence of the best and worst masterbatch alternatives on the thermal and mechanical properties of a PP/GF composite intended for the fabrication of automotive parts was also evaluated. Considering this application, the PP/GF composites containing the two masterbatches were studied for the first time by nanoindentation and nanoscratch tests.

### Significance of the Analysis

There is an increasing pressure on the plastic manufacturing industries, especially in the automotive sector, to produce high and constant quality parts. The availability of nanofillers and their usage in the manufacturing of automotive parts brought not only the possibility of obtaining improved properties but also a great challenge related to the proper dispersion of nanofillers in polymers and the proper design of the processing line and parameters. The use of masterbatches as a way to add nanofillers in polymers became widespread due to its multiple advantages. However, the process design for the manufacture of masterbatches carrying nanofillers is far from simple and well understood. A multitude of parameters should be considered to obtain an easy to disperse masterbatch in the polymer matrix, such as equipment, shear stress elements and processing parameters, to name only the most important. Our thorough analysis on the influence of halloysite nanotubes–SEBS masterbatches on PP hybrid composites for automotive parts is an attempt to understand the factors that influence the properties of the masterbatch and, further, their effect on the properties of the final composite material. This analysis was devoted to materials currently used in the automotive industry for the manufacture of crossbeams, side doors, bumpers and pillars; however, it may also serve for other industrial applications.

## 2. Materials and Methods

### 2.1. Materials

High flow polypropylene copolymer BJ380MO (PP) produced by Borealis AG (Vienna, Austria) with a melt flow index (MFI) of 80.0 g/10 min (230 °C/2.16 kg) and a density of 0.906 g/cm^3^ was used as polymer matrix. Kraton 1652G (SEBS) from Kraton Polymers (Houston, TX, USA), a linear poly[styrene-b-(ethylene-co-butylene)-b-styrene] with 29% styrene content, Mn = 79,100, density of 0.91 g/cm^3^, and MFI = 5.0 g/10 min (230 °C/5 kg) was used to manufacture the masterbatch and as an impact modifier in PP. Maleic anhydride grafted polypropylene (MAPP), Polybond 3200 from Crompton (Middlebury, CT, USA), with density = 0.91 g/cm^3^ and melting point = 157 °C served as a compatibilizer. Modified Halloysite nanotubes (HNT-QM), produced by ABC Company (Rochester, NY, USA), with kaolin content > 95%, quart < 1%, trade secret components < 5%, and a basal spacing of 7.40 Å, were used as inorganic nanofiller. Chopped glass fibers coated with silane-based sizing, ThermoFlow 636 (GF), produced by Johns Manville (Denver, CO, USA), were used as reinforcing agent.

### 2.2. Preparation of SEBS/HNT-QM Masterbatches

**Figure 1:**
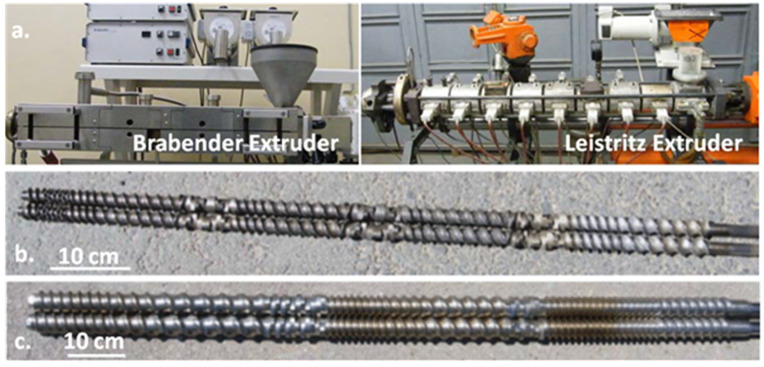
The lab-scale extruders used in the tests (**a**); screw profiles of the Brabender (**b**) and Leistritz (**c**) extruders.

The masterbatches were obtained in dynamical conditions using two co-rotating twin screw extruders: DSE 20 Brabender (Brabender GmbH & Co KG, Duisburg, Germany) and LSM 30.34 Leistritz (Leistritz Extrusionstechnik GmbH, Nürnberg, Bayern, Germany) (Figure 1a). Both pieces of equipment are laboratory co-rotating twin screw extruders, the Brabender having a shorter length than the Leistritz. The main technical characteristics of the extruders are presented in Table 1.

Besides their length, the extruders are differentiated by their screw profile. The screw diameter of the Brabender extruder is 20 mm, and the screw profile (Figure 1b) includes elements which ensure harsh mixing conditions. It contains a feeding and transport zone, a melting zone constituted of two blocks of kneading discs, one at 60° and one at −60°, and two mixing zones containing blocks of kneading discs with the stagger angle of 60° and −60° (reverse elements). Before and after each kneading block, there are left-handed elements, and before the screw end, there is an additional mixing section with tooth block elements. The high stagger angle ensures high distributive and dispersive mixing [29]. The screw diameter of Leistritz extruder is 34 mm. The melting zone (Figure 1c) contains two blocks of kneading discs with stagger angles of 30° and 60°, and the only mixing zone contains two blocks of kneading discs at 30° and 60° and two blocks of kneading discs staggered at 90°. This combination of stagger angles ensures a good balance between the mixing and conveying capacity of the kneading blocks [29].

The masterbatch components, SEBS and HNT-QM, in a ratio of 20:1, as established by a patented procedure [30], were mixed in a rotating mixer at room temperature for 30 min and then introduced in the main hopper of the extruder. Two temperature profiles from hopper to die were considered on both extruders:

*(1)* 155, 160, 175, 170, 170 and 170 (die) °C for Brabender extruder and 155, 160, 160, 160, 160, 175, 175, 170, 170 and 170 (die) °C for Leistritz extruder and

*(2)* 140, 180, 180, 180, 180 and 180 (die) °C for Brabender extruder and 140, 180, 180, 180, 180, 180, 180, 180, 180 and 180 (die) °C for Leistritz extruder.

Two screw rotation speeds (220 and 330 rpm) were tested in each case. The extruded filaments were cooled down into a water bath and then granulated using a Brabender or Leistritz granulator, which are components of the extrusion lines. The masterbatch granules were dried for 2 h at 80 °C before further processing. Square plates of 100 × 100 × 1.2 mm for thermal and mechanical characterization were obtained by hot pressing in an electrically heated press at 165 °C for 4 min, under a pressure of 12.5 MPa. After compression molding, the samples were cooled to room temperature in a cooling cassette. The masterbatches obtained with the Brabender extruder were denoted as BMB220-1, BMB220-2, BMB330-1 and BMB330-2 and those obtained with the Leistritz extruder were denoted as LMB220-1, LMB220-2, LMB330-1 and LMB330-2 considering the screw speed (220 or 330 rpm) and the temperature profile (*1* or *2*).

### 2.3. Preparation of PP Hybrid Composites

Selected SEBS/HNT-QM masterbatches, with the lowest and highest thermo-mechanical properties, were used to obtain PP/MAPP/SEBS/HNT-QM/GF composites. PP, MAPP, selected masterbatches and GF were mixed in a rotating mixer at room temperature for 30 min and then extruded in a Leistritz LSM 30.34 co-rotating twin-screw extruder at a screw speed of 220 rpm. Extruder temperature profile from hopper to die was 180, 185, 190, 195, 200, 210, 205, 200, 170 and 160 °C. The extruded filaments passed through a cooling bath and were granulated, resulting in hybrid composite granules, which were denoted as DB (with the worst masterbatch) and DL (with the best masterbatch). These were dried in an oven for 2 h at 80 °C and injection molded in standard tensile and impact strength specimens. The conditions set for the injection molding machine (Engel 40/22) were 200, 210, and 220 °C inside the barrel, the mold temperature being maintained at 50 °C. Comparing the influence of the processing conditions, the hybrid composites obtained by selected masterbatches’ dilution had a unique composition: 2.5 wt.% MAPP, 20 wt.% SEBS, 1 wt.% HNT-QM and 20 wt.% GF.

### 2.4. Characterization

#### 2.4.1. Thermo-Gravimetric Analysis (TGA)

Thermo-gravimetric analysis of masterbatches was performed on a TA-Q5000 V3.13 (TA Instruments, New Castle, DE, USA) between 25 and 700 °C at a heating rate of 10 °C/min. Nitrogen was used as the purge gas at a flow rate of 40 mL/min. Duplicate samples weighing between 8 and 10 mg were used for each measurement. PP hybrid composites were characterized using a SDT Q600 (TA Instruments, New Castle, DE, USA) under helium flow, 100 mL/min. Samples of 8–10 mg sealed in alumina pans were heated at 30 to 700 °C with 10 °C/min.

#### 2.4.2. Attenuated Total Reflectance–Fourier Transform Infrared (ATR-FTIR) Spectroscopy

The FTIR spectra were recorded on a JASCO 6300 FT-IR spectrophotometer (JASCO Int. Co., Ltd., Tokyo, Japan) equipped with a Golden Gate ATR (diamond crystal) from Specac Ltd. (London, UK). The spectra were acquired for SEBS, masterbatches and PP hybrid composites in the range of 4000–400 cm^−1^ with 30 scans per spectrum and a resolution of 4 cm^−1^.

#### 2.4.3. Dynamic Mechanical Analysis (DMA)

Dynamic mechanical analysis was performed on masterbatches using a DMA Q800 from TA Instrument (New Castle, DE, USA) with a heating rate of 3 °C/min, in tension mode. Rectangular specimens of 23 mm × 7 mm × 1.2 mm (length × width × thickness), cut from the compressed plates, were used for the measurements. Duplicate masterbatches samples were scanned over a temperature range of 30–150 °C at 1 Hz. Storage modulus (*E′*) and mechanical loss factor (tan δ) of masterbatches were plotted vs. temperature. PP hybrid composites were characterized by DMA with a heating rate of 3 °C/min, using the same DMA Q800 equipment in dual cantilever mode. Duplicate composite samples were scanned over a temperature range of 30–120 °C at a frequency of 1 Hz. Storage (*E′*) and loss (*E″*) moduli of PP and hybrid composites were plotted vs. temperature.

#### 2.4.4. Scanning Electron Microscopy (SEM)

To investigate the dispersion of modified nanotubes, SEM micrographs were taken on the surface of masterbatches using a Zeiss-Evo LS 15 environmental scanning electron microscope (ESEM) equipment (Carl Zeiss Microscopy, Dublin, CA, USA). The images were recorded on samples without covering using an accelerating voltage of 10 kV EHT and a Variable Pressure Secondary Electron Detector (VPSE G3).

#### 2.4.5. Mechanical Tests on Masterbatches and PP Hybrid Composites

##### Conventional Tensile and Impact Tests

Masterbatches were tensile tested at room temperature (23 ± 2 °C) on a universal testing machine, Instron 5944 (Instron Corporation, Norwood, MA, USA), with a 2 kN load cell and a crosshead speed of 200 mm/min. Rectangular specimens with a cross-sectional area of 4.2 mm × 1.2 mm were cut from the compression molded plates. The mean values for strength and stiffness were reported considering the results obtained in five different specimens and the error domain.

The tensile properties of the PP hybrid composites were determined according to ISO 527, at room temperature, using an Instron 3382 universal testing machine (Instron Corporation, Norwood, MA, USA). A crosshead speed of 50 mm/min was used to determine the tensile strength and of 2 mm/min to obtain the modulus of elasticity. The reported values are the average of seven individual determinations for each PP hybrid composite sample. Notched Izod impact strength of hybrid composites was measured according to ISO180, at room temperature, using a Zwick HIT5.5P (Zwick Roell AG, Ulm, Germany). Seven specimens were tested for each sample, and the mean value was reported.

##### Nanomechanical Characterization

Nanomechanical tests (nanoindentation and nanoscratch) were carried out at room temperature on a TI Premier system (Hysitron Inc., Minneapolis, MN, USA) using a three-side pyramidal Berkovich tip with a total angle of 142.35° and radius of curvature of 150 nm. High-resolution in situ scanning probe microscopy (SPM) images were recorded using the same probe as for nanomechanical tests.

Load-controlled nanoindentation tests were performed to determine the hardness (*H*) and reduced modulus (*E_r_*). Four indents were applied on each sample, using a trapezoidal load function, with maximum indentation loads of 600 µN for SEBS and SEBS based masterbatches and 10,000 μN for PP hybrid composites. All values were taken as an average of four indentations.

Three nanoscratch experiments were performed on PP and PP hybrid composites. A normal load of 5000 μN was applied in a controlled manner to the indenter tip, and the lateral force and lateral displacement were recorded as a function of time. The length of the scratches was 15 µm. After scratching, a representative 25 µm × 25 µm SPM image was obtained of each sample for post-test qualitative surface characterization and for quantitative nanoscratch analysis: coefficient of friction, cross profile topography, residual deformation of the material during the scratch, surface roughness and depth of scratch.

#### 2.4.6. Differential Scanning Calorimetry (DSC)

PP hybrid composites were characterized using a DSC Q2000 (TA Instruments, New Castle, DE, USA) under helium flow (100 mL/min). The samples were cooled to −90 °C, equilibrated for 5 min, heated to 205 °C with 10 °C/min (first heating), equilibrated for 5 min, cooled to −90 °C with 10 °C/min (cooling cycle), equilibrated for 5 min and heated again to 205 °C with 10 °C/min (second heating). The crystallization temperature (*T_c_*) was taken as the peak temperature of the crystallization exotherm (cooling cycle), and the melting temperature (*T_m_*) was taken as the peak temperature of the melting endotherm (second heating). PP crystallinity (*X_c_*) was calculated from the second heating cycle with the equation:

(1) $$X_{c}\;=\;\frac{\Delta H}{\Delta H_{0}\;w_{pp}}\;\cdot \;100$$

where Δ*H* and Δ*H*_0_ are the heat of fusion of the composite and 100% crystalline PP (207 J/g [31]) respectively, and *w_pp_* is the weight fraction of PP in the composite.

#### 2.4.7. XRD Analysis

XRD analysis of PP, HNT-QM, LMB330-1 masterbatch and hybrid composites (DB and DL) was performed on a DRON-UM X-ray diffractometer (horizontal goniometer–Bragg–Brentano geometry–reflexion mode) using Co Kα radiation with λ = 1.79021 Å. The samples were scanned at 0.05°/4 s from the 2θ value of 4° to 50°. Specimens for XRD analysis were taken from masterbatch plates 1.2 mm in thickness and 30 mm in diameter and from composites injection-molded bars of 4 mm in thickness and with a surface of 20 × 20 mm. The d spacing (d) was calculated using the Bragg equation: d = λ/(2 sinθ_max_).

## 3. Results

### 3.1. Thermo-Gravimetric Analysis (TGA) of Masterbatches

**Figure 2:**
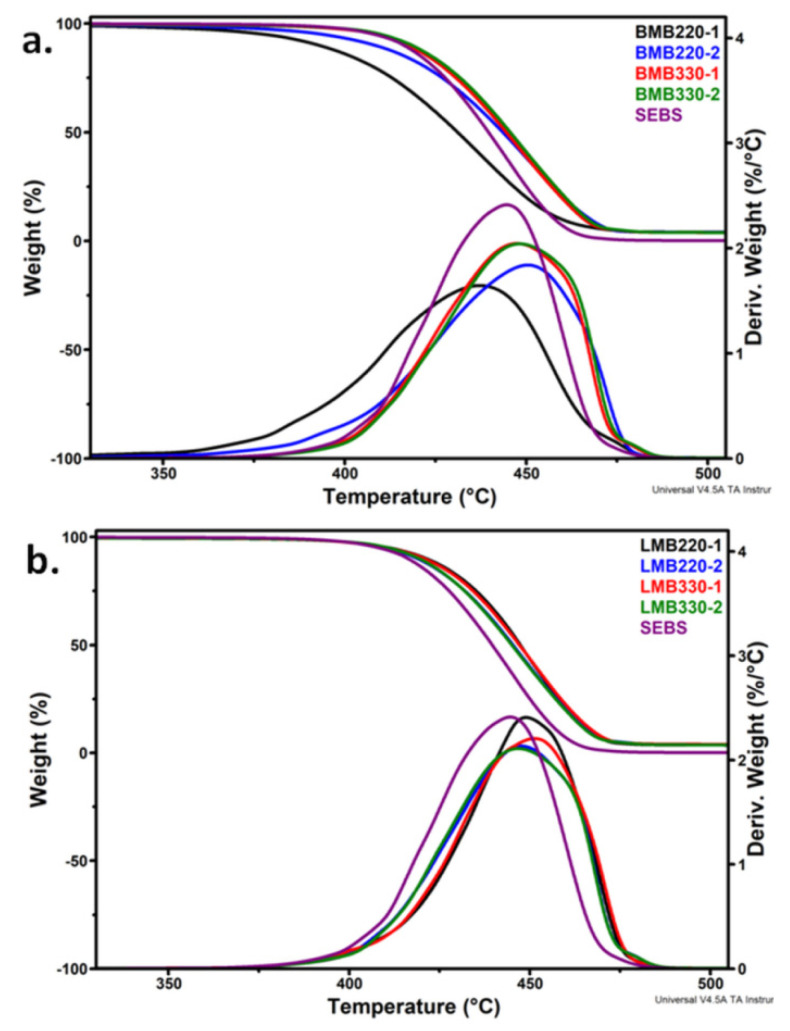
TGA and DTG curves of masterbatches obtained with Brabender (**a**) and Leistritz (**b**) extruders.

The thermal stability of SEBS/HNT-QM masterbatches may be influenced by both processing parameters (screw speed, melt temperature) and screw configuration. The results of TGA measurements on SEBS/HNT-QM masterbatches and original SEBS are presented in Figure 2. The onset decomposition temperature (*T_on_*), the temperature of the maximum decomposition rate (*T_max_*) and the residue at 700 °C (R_700_) were determined from the thermo-gravimetric curves and collected in Table 2.

Most of masterbatches had a better thermal stability than that of SEBS, the one-step weight loss curves being shifted to a higher temperature. Similar increase of thermal stability was reported for other polymer-clay nanocomposites [10,32]. In particular, the masterbatches processed in the Leistritz extruder showed a higher *T_max_* value with 2–7 °C, depending on the screw speed and melt temperature conditions (Table 2). The increased thermal stability is due to either the barrier properties of HNT, which entrap the gaseous decomposition products, or the interactions at polymer-HNT interface, induced by the melt shearing during extrusion [32]. However, the masterbatches processed in the Brabender extruder at a lower screw rotation speed (220 rpm) began to decompose at a lower temperature compared to neat SEBS, with 16 °C or 3 °C depending on the temperature profile (Table 2); as a matter of fact, only BMB220-1 had a significant lower *T_max_* value compared to the other samples (Table 2). The reduced thermal stabilities of BMB220-2 and, especially, BMB220-1 are a result of the more severe mixing conditions ensured by the Brabender extruder and the much higher residence time in the extruder at this low screw speed. These conditions led to an increased shearing and, thus, to the breakage of the elastomeric chains and further to the formation of oxidation products.

The difference between BMB220-1 and BMB220-2 was determined by the different temperature profile in the plasticization zone, with about 10 °C lower in the case of the first sample. This means an increased melt viscosity in this case and, therefore, more shear resulting in local rise in temperature and further chain breakage. Indeed, the torque values measured by the Brabender WinExt software during extrusion, 78 Nm for BMB220-1 and 74 Nm for BMB220-2, indicated a higher melt viscosity for the first sample, which is prone to degradation. Contrarily, the combination of a higher length with a lower residence time in the Leistritz extruder and the milder shearing provided by its screw profile led to an increased thermal stability in this case. This may be also associated with better nanoclay dispersion and interaction between components. The best thermal stability was noticed for LMB330-1, which showed an increase of the *T_max_* with about 7 °C compared with neat SEBS.

The theoretical value of the residue at 700 °C considering the weight losses of neat SEBS (0.14%) and HNT-QM (83.59%) is 4.11%. The lower R_700_ values in Table 2 (variations by 7–15% compared to the theoretical value) may be caused by either a poor dispersion of HNT-QM in SEBS which results in a large variation in the local concentration of HNT-QM or a good interaction between SEBS and HNT-QM which leads to the decomposition of the organic compound on the surface of HNT (probably a quaternary ammonium salt, not specified in the product data sheet) together with SEBS. The thermal lability of the organic modifier of HNT may be observed from the lower *T_on_* value, of only 310 °C.

### 3.2. FTIR Spectroscopy on Masterbatches

**Figure 3:**
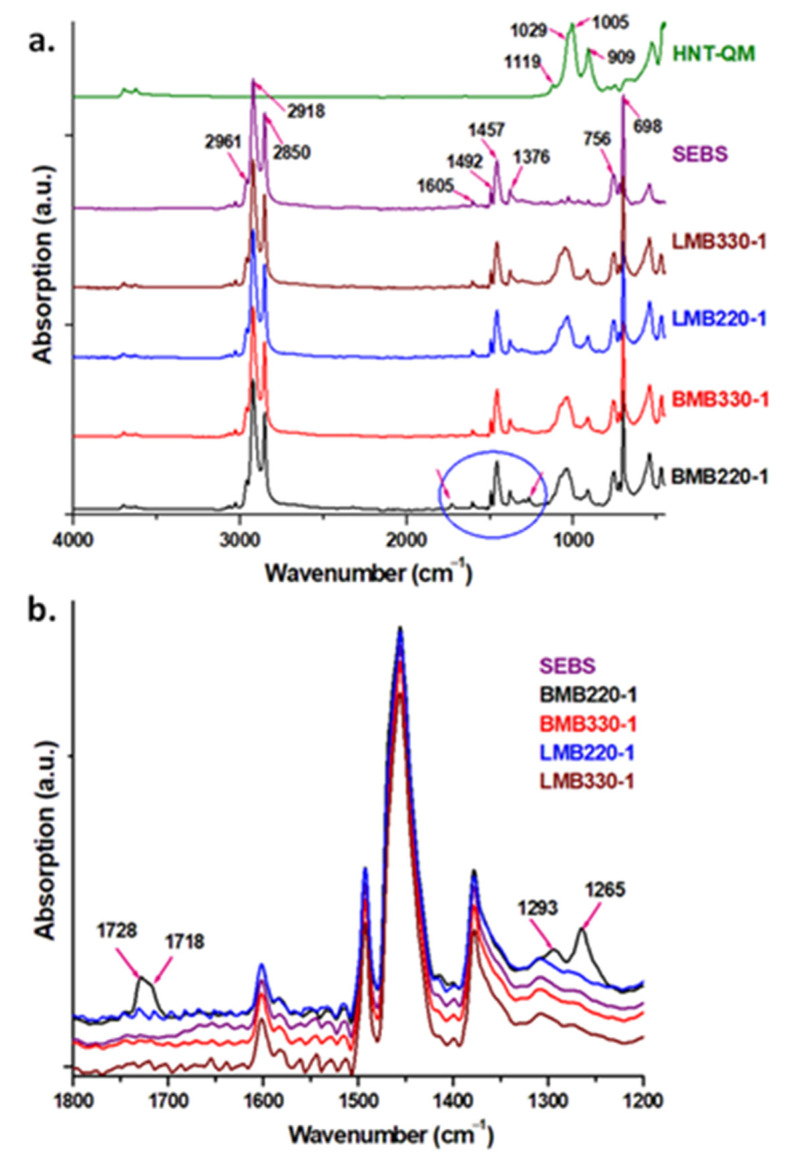
FTIR spectra for HNT-QM, SEBS and masterbatches obtained with the low temperature profile during extrusion: (**a**) full range and (**b**) narrow range 1800–1200 cm^−1^.

The differences in thermal stability between BMB220-1, the masterbatch processed in the Brabender extruder with a lower screw rotation speed at a lower temperature and the rest of the samples may result from thermo-oxidative degradation processes. Therefore, HNT-QM, SEBS and all the masterbatches obtained under the first temperature profile condition were investigated by FTIR, and the spectra are presented in Figure 3a.

The main characteristic peaks of SEBS are: the C–H bending of the monosubstituted benzene ring at 698 cm^−1^ and 756 cm^−1^ [33,34], the C–H bending in CH_3_ groups at 1376 cm^−1^ and in CH_2_ groups at 1457 cm^−1^ [33], the C=C stretching vibrations of aromatic ring at 1492 and 1605 cm^−1^ [35], symmetrical and asymmetrical stretching vibrations of the CH_2_ groups in the ethylene-butylene units at 2850 and 2918 cm^−1^ along with the asymmetrical stretching vibrations of CH_3_ groups [33]. Spectra of all masterbatches contain the bands characteristic to both SEBS and HNT. The most prominent peaks of HNT, visible in masterbatches, are located at 909 cm^−1^, 1005/1029 cm^−1^ and 1119 cm^−1^; they are ascribed to the OH deformation of inner hydroxyl groups from Al–OH, in-plane and perpendicular Si-O stretching vibrations, respectively [5,36]. In the FTIR spectra of all masterbatches, a blue shift was noticed for Si–O stretching vibrations, which may be an effect of SEBS–HNT interactions [36].

Figure 3b displays the FTIR spectra of the masterbatches obtained with different screw speed and profile in the interval 1800–1200 cm^−1^. Compared to the other samples, the masterbatch processed at 220 rpm in the Brabender extruder exhibits new FTIR peaks. The occurrence of the bands at 1718 cm^−1^ and 1728 cm^−1^, assigned to the C=O stretching of carboxylic acids and straight chain esters [23], respectively, implies the formation of oxidation products. Furthermore, the two peaks at 1265 cm^−1^ and 1293 cm^−1^ could be also attributed to C–O stretching of esters [37]. The appearance of degradation products during extrusion is an effect of the longer residence time at a lower screw speed and of the higher viscosity at a lower temperature profile during extrusion, both resulting in an increased shear of the polymer melt. Therefore, the FTIR results clearly emphasize the degradation of BMB 220-1 SEBS/HNT masterbatch, in good agreement with the lower characteristic temperatures obtained by TGA for this sample (Table 2).

### 3.3. Dynamic Mechanical Analysis (DMA) of Masterbatches

**Figure 4:**
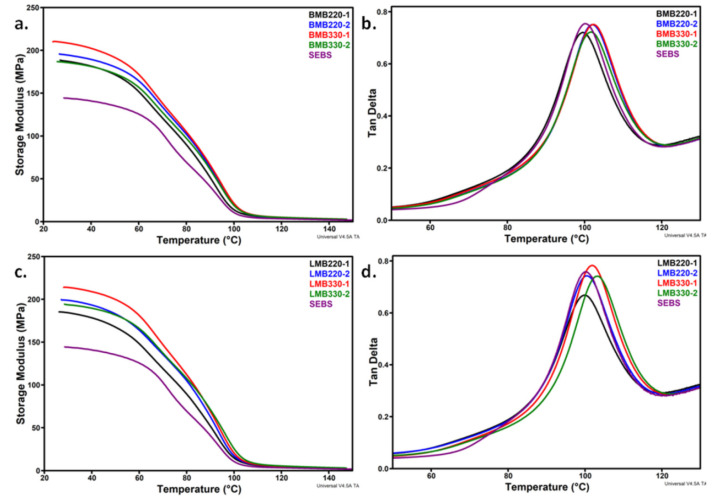
Storage modulus vs. temperature for masterbatches obtained with Brabender (**a**) and Leistritz (**c**) extruders; loss factor vs. temperature for BMB (**b**) and LMB (**d**) samples.

The higher thermal stability of SEBS/HNT-QM masterbatches than that of neat SEBS, which was observed in almost all the tested conditions, could be ascribed to a direct stabilizing effect of HNT on SEBS based on increased interactions. This was suggested by FTIR results and is similar to other observations [32]. The possible enhanced interactions between the two components may be assessed by the investigation of the molecular mobility using DMA. Figure 4 presents the storage modulus (*E′*) and tan δ as functions of temperature for masterbatches processed in the Brabender or Leistritz at a different screw speed (220 rpm or 330 rpm) using the two temperature profiles. Table 3 lists the storage modulus at 30 °C and 100 °C and the glass transition of the hard PS domains (*T_g_*).

All the masterbatches showed a higher storage modulus compared to SEBS on the whole temperature range. This is due to the reinforcing effect of HNT also signaled in the case of other polymer matrices [20]. Since the stiffening effect of HNT is favored by a good dispersion of nanotubes in SEBS and enhanced interfacial interactions, a higher increase of *E′* can be attributed to a better dispersion and increased interactions between phases. The highest increase of *E′*_30_, between 35% and 48% compared to SEBS, was noticed in the same conditions for both Brabender and Leistritz processed masterbatches, either lower screw speed and higher temperature (BMB220-2, LMB220-2) or higher screw speed and lower temperature (BMB330-1, LMB330-1) (Table 3). It is known that, on the one hand, the specific mechanical energy input and the temperature in the melt increase at a higher screw speed, and on the other hand, a lower set-temperature leads to a higher melt viscosity of the polymer and an increased specific mechanical energy input [38,39]. Therefore, the two factors, screw speed and temperature, can be interchanged leading to a higher energy input and better mixing of components, with effect on the dispersion of nanofiller and interactions between phases. It is remarkable that the highest *E′*_30_ (close to room temperature) was obtained for low temperature profile–high screw speed conditions, regardless the extruder.

The *T_g_* of the polystyrene (PS) blocks of SEBS was determined from the tan δ curve vs. temperature (Figure 4b,d). Previous work has shown that the glass transition of ethylene–butylene block of SEBS is not influenced by the addition of a nanosilicate, which increases only the *T_g_* of PS blocks [40]. Contrarily, a slight decrease of the *T_g_* of PS blocks, showing weaker interactions at the polymer/filler interface, was reported for SEBS/graphite composites [41]. In the case of SEBS/HNT-QM masterbatches, the *T_g_* values were around 100 °C. An increase with 2–3 °C was noticed in several cases (Table 3). In particular, the samples processed in Brabender and characterized by the highest *E′* values (BMB220-2, BMB330-1) also presented increased *T_g_* values. Therefore, these conditions ensure better mixing and interactions between HNT and the PS blocks of SEBS.

In the case of the Leistritz processed samples, only those obtained with a higher screw speed showed increased *T_g_* values. The highest increase was obtained for LMB330-2, processed under the harsher conditions of the Leistritz. These two samples, LMB330-1 and LMB330-2, also showed the highest *E′*_100_ values and higher storage modulus for a wide range of temperature (Figure 4c). These differences result from the different mixing conditions ensured by the two extruders, the more severe ones in the case of the Brabender extruder.

### 3.4. Tensile Properties of SEBS/HNT-QM Masterbatches

**Figure 5:**
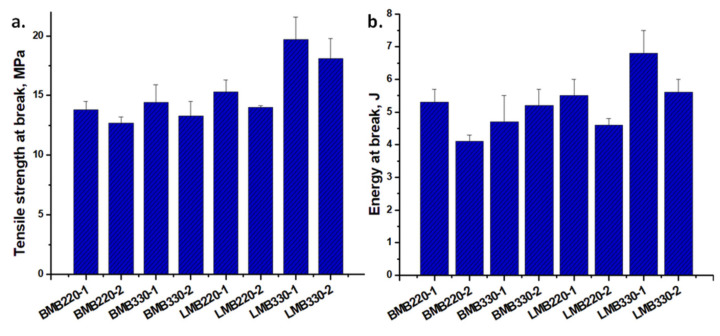
Tensile strength (**a**) and energy at break (**b**) for masterbatches processed in the Brabender or Leistritz extruders.

The influence of processing conditions on the mechanical properties of polymer–clay nanocomposites was scarcely studied. Higher storage modulus and impact strength were reported for a medium shear intensity configuration of the screw in the case of a PP-clay nanocomposite [42]. Similarly, the reinforcing effect of organoclay measured by the increase of the storage modulus in PS-organoclay nanocomposite was effective only at a low screw speed in the case of a more aggressive screw profile [43]. No information on the cumulative influence of screw profile, screw speed and temperature was reported yet. Tensile strength and energy at break of masterbatches processed on the two extruders in different conditions are presented in Figure 5.

As a rule, the tensile strength of SEBS/HNT-QM samples processed in the first temperature condition (low temperature profile) is slightly higher than that of the samples processed with the second higher temperature profile. A lower extrusion temperature results in a higher melting viscosity and more intense shear, leading to a better dispersion of the nanofiller [21,22,24] and a higher tensile strength. For the masterbatches processed in the Brabender extruder, the screw speed had no influence on the tensile strength; however, a higher screw speed led to increased tensile strength when the masterbatches were processed in the Leistritz. These differences were caused by the different screw profiles of the two extruders; the more severe mixing conditions ensured by the Brabender extruder reduced the favorable influence of the screw speed, which was observed only in the case of the milder shearing provided by the Leistritz extruder. Similarly, using two screw profiles on a Leistritz twin-screw extruder and fixed operating conditions (100 rpm and 200 °C), Barbas et al. observed a more drastic decrease of the final yield stress of the PP/organoclay nanocomposite in the case of the more severe screw profile [44].

The same rule was noticed for the energy at break, higher values being obtained for the lower temperature profile in the case of the masterbatches processed in the Leistritz and no clear variation for those processed in the Brabender. The highest tensile strength and energy at break values were obtained for the LMB330-1 masterbatch, processed with a higher screw speed and a lower temperature profile, which also showed the best storage modulus and the best thermal stability.

### 3.5. Nanoindentation Tests on Masterbatches

**Figure 6:**
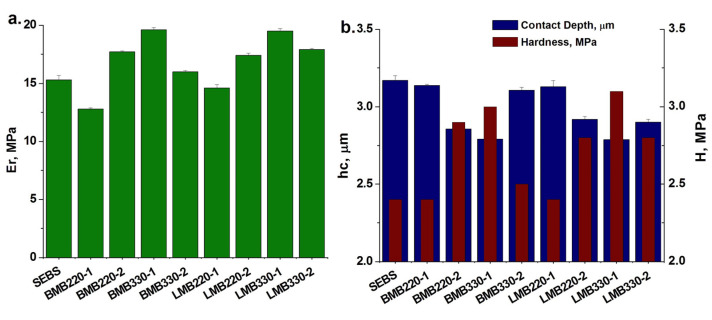
Reduced modulus (*E_r_*) (**a**), hardness (*H*) and contact depth (*h_c_*) (**b**) of masterbatches processed in Brabender and Leistritz extruders in comparison with SEBS.

Nanoindentation tests were also carried out on SEBS/HNT-QM masterbatches due to the great importance of the surface properties in automotive applications. The variation of *E_r_* and *H* for masterbatches in comparison with SEBS is presented in Figure 6. A similar trend was observed for the *E_r_* values measured by nanomechanical test (Figure 6a) and *E′* measured by DMA (Figure 4a,c), namely, an increased modulus for the masterbatches processed at high speed and low temperature profile. The highest increases in *E_r_* (of about 28%) compared to SEBS were obtained for the masterbatches processed at 330 rpm and low temperature, in both Brabender and Leistritz (BMB330-1and LMB330-1). The lowest value was obtained for BMB220-1, the masterbatch processed at 220 rpm and low temperature, which also showed the most intense degradation during processing.

The hardness correlates well with the reduced modulus and also with the contact depth, a lower hardness corresponding to a greater contact depth (Figure 6b). The softness of the samples’ surface was evaluated from the values of maximum displacement (*h_max_* at peak load of 600 μN) and residual depth after final unloading (final depth—*h_f_*) (Table 4). The BMB220-1 and LMB220-1masterbatches are the “softest” samples, showing the highest degree of elastic recovery during unloading, while the “hardest” are LMB330-1 and BMB330-1, in good agreement with the tensile tests results.

### 3.6. SEM Analysis of Masterbatches

**Figure 7:**
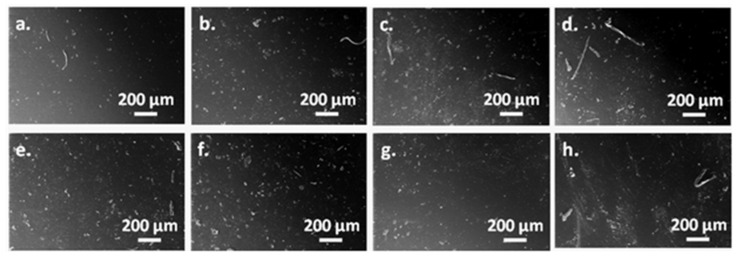
SEM images of masterbatches obtained with the two extruders: BMB220-1 (**a**), BMB220-2 (**b**), BMB330-1 (**c**), BMB330-2 (**d**), LMB220-1 (**e**), LMB220-2 (**f**), LMB330-1 (**g**), LMB330-2 (**h**).

The dispersion of nanotubes in a layer of a couple of nanometers on the surface of SEBS/HNT-QM masterbatches was analyzed by SEM. Figure 7 shows representative SEM images of masterbatches obtained with the two extruders under different conditions. Only the nanotubes agglomerations are visible at this magnification, identical for all the images. The presence of nanotubes agglomerations of micrometric and submicrometric size is observed in all the images. Regardless the screw profile, the worse dispersion was observed for BMB330-2 and LMB330-2 (Figure 7d,h). The samples were obtained under the same conditions: the highest screw speed and temperature profile. It is interesting that they also showed a lower storage modulus (Table 3) and tensile strength (Figure 5). Therefore, the harsher shear and temperature mixing conditions do not lead to a good dispersion of nanotubes and better mechanical properties. Similar conclusions were reported for other nanocomposite systems [22,42,43].

**Figure 8:**
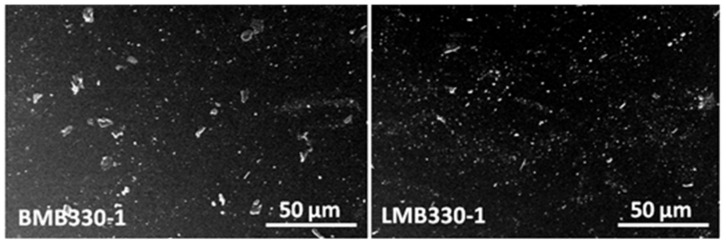
SEM images of BMB330-1 and LMB330-1.

The two SEBS/HNT-QM masterbatches with the best mechanical and thermal properties, BMB330-1 and LMB330-1, were investigated in more detail (Figure 8). Both showed a good dispersion of nanotubes and small size agglomerations, of 1–5 µm in BMB330-1 and much smaller, less than 1 µm, in the case of LMB330-1, in agreement with the better properties of this last sample.

Therefore, a higher screw speed and lower temperature profile correlated with a mild-shear longer screw may ensure the best mixing conditions for the SEBS/HNT-QM masterbatches. The masterbatches with the best and worst thermal and mechanical properties, LMB330-1 and BMB220-1, were selected for further tests as masterbatches in PP hybrid composites.

### 3.7. Thermal, Mechanical and Structural Properties of PP Hybrid Composites

**Figure 9:**
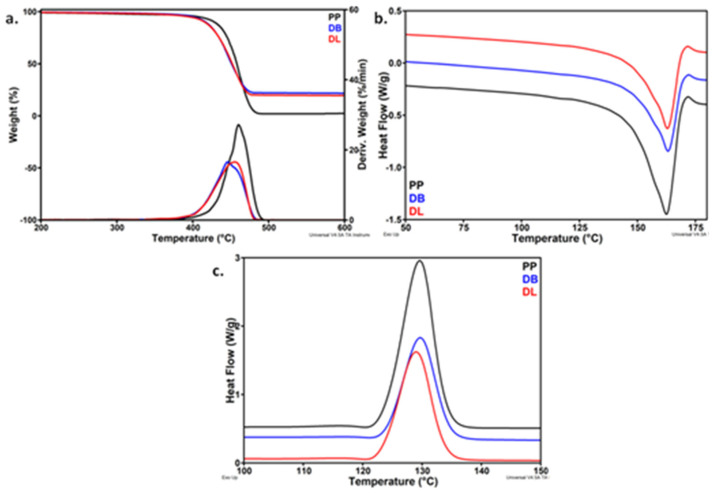
TGA-DTG curves (**a**), DSC heating (**b**) and cooling (**c**) cycles of PP and PP hybrid composites.

The effect of masterbatch quality on the properties of a PP hybrid composite was studied by diluting the selected masterbatches, BMB220-1 and LMB330-1, in a PP/GF composite also containing MAPP, according to a manufacturing recipe for automotive parts [30]. The Leistritz LSM 30.34 co-rotating twin-screw extruder was used for the dilution due to its mild configuration, which protects GF from severe breaking. The thermal properties of hybrid composites obtained by dilution of BMB220-1 and LMB330-1, denoted as DB and DL, were compared to those of the original PP (Figure 9).

Both composites began to decompose at a lower *T_on_*, by up to 20 °C, compared to pristine PP; the maximum degradation temperature was 5 °C lower in the case of DL and 15 °C in the case of DB (Table 5). Therefore, the more severe mixing conditions ensured by the Brabender extruder were reflected in the decreased thermal stability of the hybrid composite, probably due of the degradation products from the masterbatch, emphasized by FTIR. It is worth mentioning that the weight loss at 300 °C (*WL*_300_), a temperature higher than that of extrusion and injection molding of automotive parts, is very low in both composites, showing a good thermal stability during the manufacture of parts. The high residue obtained for hybrid composites was determined by the presence of GF and HNT-QM with a high thermal stability up to 700 °C [45].

The DSC results of the cooling and second heating cycles (Figure 9b,c and Table 5) showed no important changes in the melting and crystallization temperatures. A slight increase of crystallinity was observed in hybrid composites compared to PP, the highest increase being observed for the DL composite. However, a decrease of the initial slope of the crystallization exotherm was noticed in hybrid composites compared to PP, from 0.57 to 0.34 and 0.37 J/°C, indicating a lower rate of nucleation [46]. Therefore, the thermal behavior of hybrid composites is a cumulative effect of nanoparticles, MAPP, SEBS and GF which influence the PP chains mobility and the nucleation and crystallization process [7].

The hybrid composites presented almost double tensile strength and Young’s modulus compared to pristine PP due to the influence of GF and HNT-QM along with SEBS. The tensile strain at break was also more than double that of PP due to the presence of SEBS toughening agent. In addition, a four-times increase of the impact strength was obtained with both hybrid composites compared to PP. The concomitant improvement of strength, stiffness and toughness is a result of the masterbatch method used to obtain the hybrid composites and of the design of compounding conditions to obtain the masterbatch [21,22]. It is worth mentioning the higher Young’s modulus and impact strength of the hybrid composite prepared with the best Leistritz masterbatch. These results support a more uniform dispersion of the masterbatch and, implicitly, of the nanosilicate in the PP matrix. The differences between the properties of the two composites may increase over time due to degradation products observed by FTIR in the BMB220-1 masterbatch.

**Figure 10:**
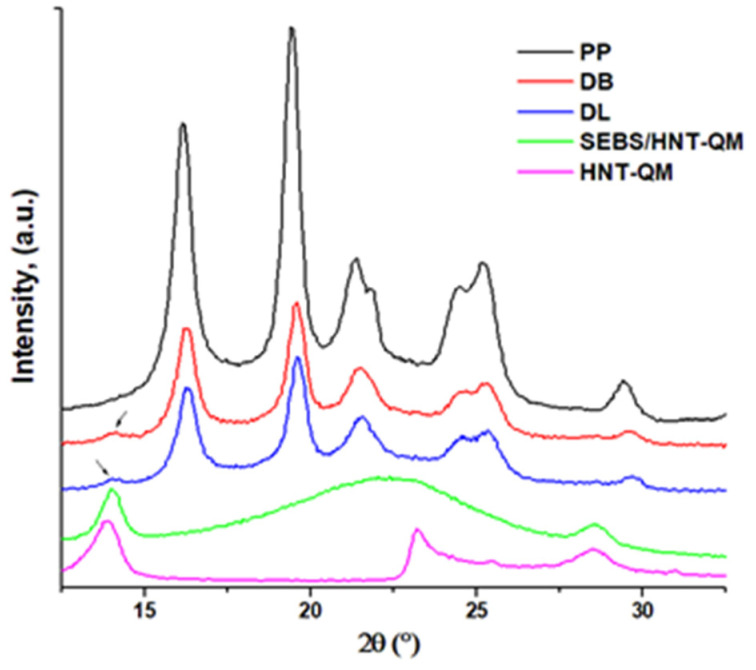
X-ray diffraction patterns of PP, HNT-QM, masterbatch and hybrid composites.

Figure 10 shows the X-ray diffraction patterns of PP hybrid composites compared to neat PP. The diffraction patterns of SEBS/HNT-QM and HNT-QM were also added for comparison. Five peaks were observed at 2θ = 16.14, 19.43, 21.34, 24.45 and 25.25° in PP and hybrid composites, and they correspond to the (110), (040), (130), (111) and (041) crystallographic plans of the α-form of PP [7]. The addition of SEBS-QM masterbatches in PP composites did not change the crystalline structure, similar patterns being obtained in the hybrid composites. A peak at 2θ = 13.9°, corresponding to HNT-QM (d = 7.4 Å), was clearly observed in SEBS/HNT-QM and as a small hump in PP hybrid composites due to its small concentration (1 wt.%) (Figure 10). The shoulder observed in PP at 2θ = 21.87° and hardly visible in hybrid composites may come from the additives used in PP which is a commercial product.

**Figure 11:**
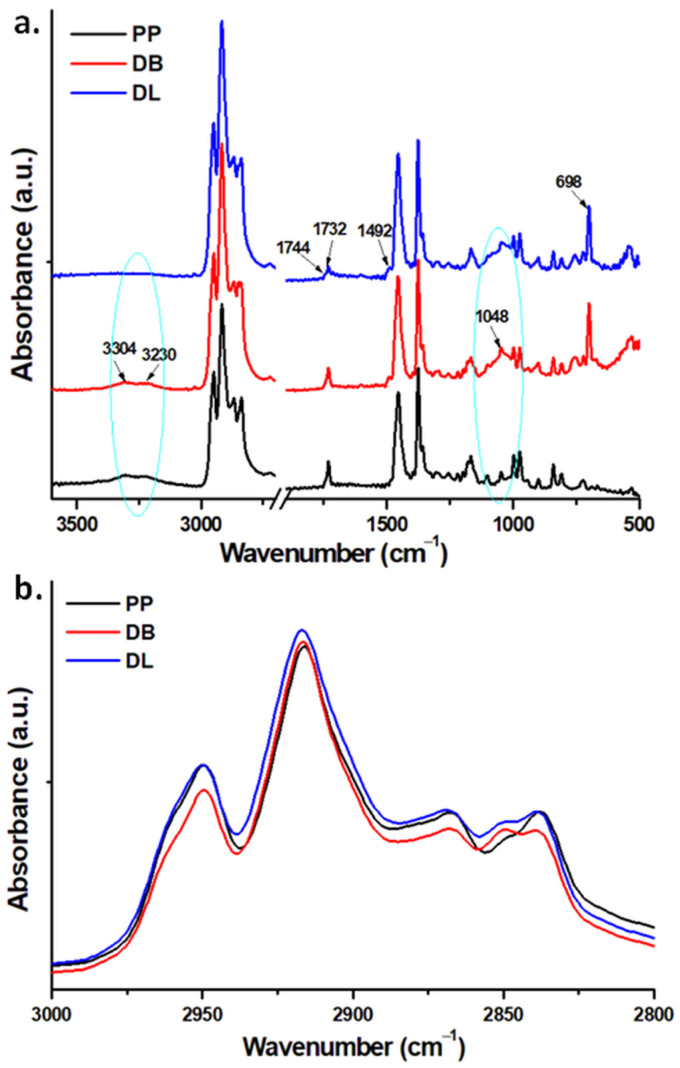
FTIR spectra of PP and hybrid composites DB and DL (**a**); normalized spectra of PP, DB and DL between 3000 and 2800 cm^−1^ (**b**).

FTIR spectra of the hybrid composites are shown in Figure 11a. The occurrence of peaks at 3304 cm^−1^ and 3230 cm^−1^ in PP may be associated with surface-bound fatty acid esters slip agents which normally are present in commercial PP products as additives that control friction [47]. This structural particularity is sustained by the presence of a carbonyl peak which appears as a twin peak with the major absorption at 1732 cm^−1^ and a shoulder at 1744 cm^−1^. Usually, a carbonyl functionality in polyolefins may arise from thermo-oxidative or photochemical degradation, as well as from the presence of additives based on esters, which is probably the case with DB and DL As a remark, in the spectrum of DL, these peaks almost disappeared, while the carbonyl stretching vibration at 1732 cm^−1^ becomes broader and smaller. This may result from several causes which will be further analyzed.

An important zone of the PP composites spectra is found at 2960–2830 cm^−1^, where the peaks are sensible to interactions of additives with PP. In detail, the peak at 2959 cm^−1^ is attributed to C–H asymmetric stretching in CH_3_ (in-skeletal plane), the peak at 2950 cm^−1^ to C–H asymmetric stretching in CH_3_ (out-of-skeletal plane), the peak at 2917 cm^−1^ to C-H asymmetric stretching in CH_2_, the peak at 2868 cm^−1^ to C–H symmetric stretching in CH_3_, the peak at 2850 cm^−1^ to C–H symmetric stretching in CH_2_ and the peak at 2838 cm^−1^ to C–H stretching in CH [48] (Figure 11b). Several differences were observed between the spectra of DB and DL in this region (Figure 11b). Variations in the intensity of the peaks in the region 2960–2830 cm^−1^ can be used as indicative for PP-SEBS interactions, especially due to a lower depth of light penetration on the ATR-FTIR, of about only 0.66 µm at 3000 cm^−1^. The higher intensities of all the peaks recorded for DL compared to DB in the analyzed region suggest the presence on the surface of DL of a certain amount of SEBS dissolved or co-crystallized with PP. This can be interpreted as a better compatibility and homogeneity of components (SEBS and PP) in DL compared with DB.

In the spectra of the composites, besides the infrared absorption bands characteristic of polypropylene in the range 1500–800 cm^−1^, the following were also noticed: a strong peak at 1377 cm^−1^ followed by a shoulder at 1360 cm^−1^, attributed to CH_3_ symmetric bending and CH_2_ wagging, bands at 1256 cm^−1^ (CH bending + CH_2_ twisting + CH_3_ rocking), 1220 cm^−1^ (CH_2_ twisting + CH bending + C–C chain stretching), 1168 cm^−1^ (C–C chain stretching + CH_3_ rocking + CH bending), 1102 cm^−1^ (C–C chain stretching + CH_3_ rocking + CH_2_ wagging + CH twisting + CH bending), 1047 cm^−1^ (C–CH_3_ stretching + C–C chain stretching + C–H bending), 998 cm^−1^ (CH_3_ rocking + CH_2_ wagging + CH bending), 973 cm^−1^ (CH_3_ rocking + C–C chain stretch), 941 cm^−1^ (CH_3_ rocking + C–C chain stretch), 900 cm^−1^ (CH_3_ rocking + CH_2_ rocking + CH bending), 841 cm^−1^ (CH_2_ rocking + C–CH_3_ stretching) and 807 cm^−1^ (CH_2_ rocking + C–C stretching + C–H stretching) [48], a shoulder at 1492 cm^−1^ due to C=C stretching vibrations of aromatic ring of SEBS. Other characteristic bands of the additives are the band at 1048 cm^−1^ corresponding to Si–O–Si asymmetric stretching vibration of the glass fibers and the peaks characteristic to SEBS (see 3.2) which were found unchanged [33].

### 3.8. Dynamic Mechanical Properties of PP Hybrid Composites

**Figure 12:**
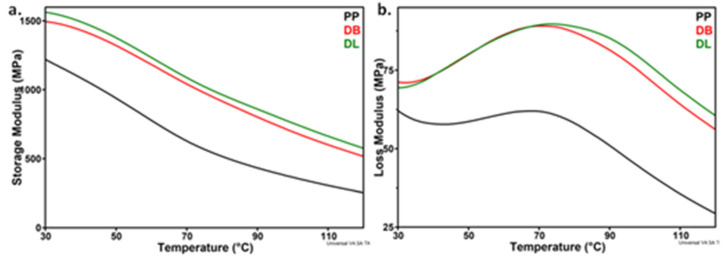
Storage modulus (**a**) and loss modulus (**b**) vs. temperature for PP hybrid composites, DB and DL, compared with neat PP.

The variation with temperature from room temperature to 120 °C of the storage modulus (*E′*) and loss modulus (*E″*) of PP and composites (DB and DL) is presented in Figure 12. Both hybrid composites showed increased *E′* values compared to neat PP on the whole range of tested temperatures, which is correlated with their higher stiffness. The greatest value of the storage modulus was obtained for the DL composite prepared by the dilution of LMB330-1 masterbatch, similar to the variation of Young’s modulus (Table 6).

A relaxation process (α-transition) was observed around 70 °C in all the samples (Figure 12b). This is associated to the molecular motion of the PP chains in the crystalline phase [49]. The *E″* peak was wider and shifted to a higher temperature in PP hybrid composites compared to neat PP (Figure 12b): 70.7 °C for DB and 73.8 °C for DL compared to 67.9 °C for PP. This supports the stronger interactions among the components which restricts the polymer chains mobility in DL composite [49,50]. This is consistent with the higher values obtained for the modulus of elasticity and impact strength of DL (Table 6).

### 3.9. Nanomechanical Properties of PP Hybrid Composites

#### 3.9.1. Nanoindentation Test Results

**Figure 13:**
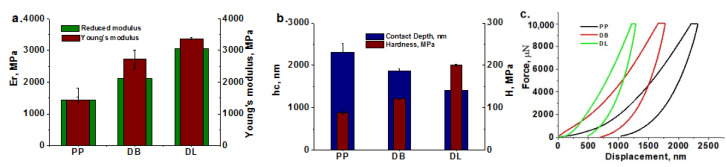
Reduced modulus vs. Young’s modulus (**a**) hardness (**b**) and load–displacement plots (**c**) for PP and PP hybrid composites obtained from selected masterbatches.

Non-destructive nanoindentation tests were carried out on hybrid composites for simultaneously highlighting the influence of micro- and nano-filers on their surface mechanical properties [51]. A comparison of the reduced modulus obtained by nanoindentation and the Young’s modulus obtained by tensile tests is shown in Figure 13a. A good correlation was noticed, the DL composite, prepared by the dilution of LMB330-1 masterbatch, showing the best modulus value. The composite DL also presents the highest hardness (Figure 13b,c). The differences between the nanomechanical properties of the two hybrid composites (DL and DB) may come from the presence of thermo-oxidative degradation products in the BMB masterbatch pointed out by FTIR or from the different dispersion and size of the HNT nanofiller, also highlighted by SEM.

These results support the essential role of homogeneity and properties of the masterbatch on the performance of hybrid composites. Moreover, they show the ability of nanoindentation tests to highlight the different effect of masterbatches.

#### 3.9.2. Nanoscratch Test Results

**Figure 14:**
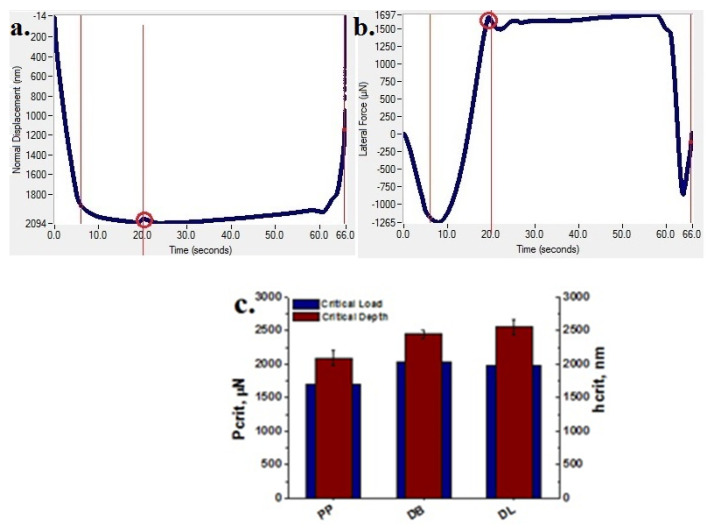
Representative plots of normal displacement (**a**) and lateral force (**b**) versus time from a 5000 μN ramping force nanoscratch test on PP. Critical events in the data (showing point of distinct material piled up) are circled and correspond to hcrit and Pcrit; Pcrit and hcrit data from 5000 μN ramping force nanoscratch tests on PP and PP hybrid composites (**c**).

Representative plots of normal displacement and lateral force versus time, generated by the TriboScan software from 5000 μN ramping force nanoscratch tests, are shown in Figure 14a,b. Critical events, defined as critical load (Pcrit) and critical depth (hcrit), were identified from these data plots. They correspond to changes in the displacement/lateral force curves (circled in Figure 14a,b).

Pcrit and hcrit data from 5000 μN ramping force nanoscratch tests, performed on PP and PP hybrid composites, are shown in Figure 14c. The highest Pcrit and hcrit values were obtained for the composite DL, prepared by the dilution of LMB330-1 masterbatch, which is considered to have the best scratch resistance properties [52].

**Figure 15:**
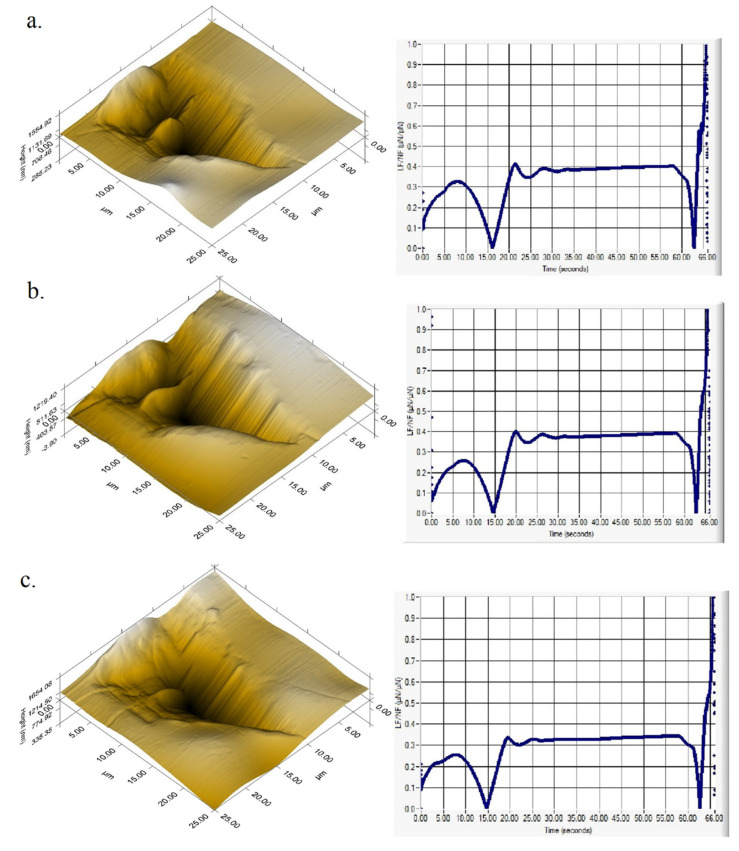
3-D topographical in situ SPM images (top) and the apparent friction coefficient (bottom), defined as the ratio between the lateral force and the applied load, obtained on PP (**a**), DB composite (**b**) and DL composite (**c**).

These results are consistent with the roughness and friction parameters measured on the surface of hybrid composites and PP: root mean square (RMS) roughness (Rq), coefficient of friction (µ), scratch depth (SD) and scratch pile-up. The height of the pile-up formed during the scratching of the samples’ surface depends on the hardness and elastic modulus of the sample: the higher the hardness and the elastic modulus of the sample, the lower is the pile-up [51]. The 3-D topographical in situ SPM images and the values of Rq, μ, SD and scratch pile-up (rear pile-up) for PP and PP hybrid composites are presented in Figure 15 and Table 7.

The topographic images show a different scratching behavior for the three samples. The samples differ by the depth of the scratch and the height of the rear pile-up residue. The SPM surface topography measurements results indicate a ±5% variation of composites’ roughness compared to PP. A decrease in the scratch depth by 24% in the case of DB composite and 40% in the case of DL composite were also obtained. The coefficient of friction is almost unchanged in the case of DB composite compared to original PP but decreases by about 20% in the case of DL composite. The lowest height of the pile-up and of the values for surface roughness, coefficient of friction and scratch depth were obtained for DL composite, prepared by the dilution of LMB330-1 masterbatch (Table 7). The better scratch resistance of this sample compared to that of DB composite is due to the higher quality of the LMB330-1 masterbatch. Therefore, the nanoscratch tests better emphasize the different efficiency of masterbatches in the PP hybrid composite. For automotive applications, the scratch resistance is as important as the rigidity, strength and toughness of PP based materials, and therefore, special attention should be paid to the processing technology and masterbatch preparation.

## 4. Conclusions

The process design for the manufacture of masterbatches carrying nanofillers in polymer matrices is far from being simple and well understood. This study is a thorough analysis of the influence of halloysite nanotubes–SEBS masterbatches on PP hybrid composites for automotive parts. Furthermore, this is an attempt to understand the factors that influence the properties of the masterbatch and, further, their effect on the properties of the final composite material. From the multitude of factors that control the dispersion of the HNT-QM in the SEBS matrix during extrusion, the influence of the screw geometry and processing parameters (temperature and screw speed) was studied here. Several correlations between the processing conditions and the properties of the masterbatches and final PP hybrid composites were observed:

1. The SEBS masterbatches showed, in general, a better thermal stability than SEBS, except for that obtained in more severe mixing conditions, ensured by the configuration of the screws, and low temperature profile set on the twin-screw extruder, which led to a higher residence time; in these conditions, the appearance of degradation products was confirmed by the FTIR analysis.

2. Better mechanical properties, determined by DMA, tensile and nanoindentation tests on masterbatches, were obtained for low temperature profile–high screw speed processing conditions, regardless of the configuration of the extruder; these two factors (low temperature, high screw speed) lead to a higher energy input and better mixing of components, with effect on the dispersion of nanofiller and interactions between phases; on the contrary, a low energy input results in a poor dispersion of HNT-QM and the degradation of SEBS; however, severe mixing conditions (high temperature and shear) worsen the dispersion of HNT-QM with effect on the mechanical properties of the masterbatch.

3. The use of SEBS/HNT-QM masterbatches with the best and worst thermal and mechanical properties, LMB330-1 and BMB220-1, in PP composites resulted in hybrid composites with much better mechanical properties, measured at nano and macro scale, compared to PP: double tensile strength and Young’s modulus values and four times higher impact strength along with improved scratch resistance; however, the thermal stability, crystallinity, storage modulus and tensile and nanoindentation moduli along with the impact strength of hybrid composites obtained with the best masterbatch were also better than in the case of the composites containing the BMB220-1 masterbatch; therefore, the PP hybrid composite with the best properties was obtained by diluting the masterbatch processed on an extruder which ensures mild shearing conditions.

Our approach provides an efficient and objective way to quantify the effect of processing conditions on the properties of nanomaterials used as masterbatches in PP composites. These results are of particular practical importance for automotive manufacturers and may serve to optimize the manufacturing process of different parts such as crossbeams, side doors and bumpers; however, they may also serve for other industrial applications.

## Figures and Tables

**Table 1:** Technical characteristics of Brabender and Leistritz extruders.

Characteristics	Brabender Extruder	Leistritz Extruder
Cylinder (Barrel) Length, mm	800	1080
The length of each heating zone, mm	182	120
Feeding zone length, mm	70	120
Number of heating zones per cylinder (barrel)	4	8
Total length of the extrusion head, mm	72	260
Extrusion dies diameter, mm	3	4
Residence time at 220 rpm, s	140–150	60–65
Residence time at 330 rpm, s	85–90	40–45

**Table 2:** Characteristic temperatures and residue at 700 °C for SEBS, HNT-QM and masterbatches.

Sample	*T_on_* (°C)	*T_max_* (°C)	R_700_ (%)
BMB220-1	402.6	437.5	3.78
BMB220-2	416.0	449.2	3.74
BMB330-1	420.2	447.6	3.69
BMB330-2	421.3	448.3	3.73
LMB220-1	424.4	449.0	3.48
LMB220-2	422.1	447.5	3.73
LMB330-1	424.6	451.2	3.80
LMB330-2	421.7	446.8	3.57
SEBS	418.7	444.7	0.14
HNT-QM	310.0	479.3	83.59

**Table 3:** Storage modulus at 30 °C (*E′*_30_) and at 100 °C (*E′*_100_) and the glass transition temperature (*T_g_*) for SEBS and masterbatches.

Sample	(*E′*_30_) (MPa)	(*E′*_100_) (MPa)	*T_g_* (°C)
BMB220-1	187.2	14.0	99.5
BMB220-2	194.6	19.8	102.0
BMB330-1	208.5	21.4	102.0
BMB330-2	185.7	19.1	101.4
LMB220-1	184.3	14.8	99.6
LMB220-2	200.1	17.4	100.2
LMB330-1	213.8	20.7	101.7
LMB330-2	193.6	25.8	102.9
SEBS	144.1	10.7	99.9

**Table 4:** The residual depth after final unloading (*h_f_*) for SEBS and SEBS/HNT-QM masterbatches.

Sample	SEBS	BMB 220-1	BMB 220-2	BMB 330-1	BMB 330-2	LMB 220-1	LMB 220-2	LMB 330-1	LMB 330-2
*h_ma__x_*, nm	4812 ± 32	5105 ± 8	4412 ± 18	4111 ± 17	4666 ± 21	4853 ± 40	4460 ± 21	4225 ± 9	4386 ± 20
*h_f_*, nm	1340 ± 32	1104 ± 8	1167 ± 18	941 ± 17	1360 ± 21	1313 ± 40	1133 ± 21	1095 ± 9	1195 ± 20

**Table 5:** TGA and DSC data for PP and PP hybrid composites obtained with the selected masterbatches.

Sample	*T_on_* (°C)	*T_max_* (°C)	*WL_300_* (%)	*R_700_* (%)	*T_m_* (°C)	∆*H_m_* (J/g)	*T_c_* (°C)	∆*H_c_* (J/g)	*X_c_* (%)
PP	440.3	460.2	2.1	2.0	162.5	98.4	129.6	98.6	47.5
DB	421.7	445.6	1.3	21.4	163.1	60.4	129.7	59.9	51.6
DL	423.4	455.4	1.6	19.1	162.9	64.0	129.0	64.0	54.7

**Table 6:** Mechanical properties of PP and PP hybrid composites obtained with the selected masterbatches.

Sample	Tensile Strength at Break MPa	Tensile Strain at Break %	Young’s Modulus MPa	Energy at Break J	Izod Impact Strengthk J/m^2^
PP	26.3 ± 0.5	3.7 ± 1.0	1455 ± 91	1.4 ± 0.5	4.8 ± 0.4
DB	41.8 ± 1.1	8.0 ± 0.6	2727 ± 292	5.8 ± 0.3	19.0 ± 1.5
DL	41.0 ± 0.6	8.5 ± 0.3	3373 ± 32	6.0 ± 0.4	21.0 ± 1.0

**Table 7:** The RMS roughness (Rq), the coefficient of friction (µ), the scratch depth (SD) and the scratch pile-up measured for PP and PP hybrid composites.

Sample	Rq (nm)	μ	SD (nm)	Rear Pile-Up (nm)
PP	207 ± 0.01	0.40 ± 0.015	902 ± 35	650 ± 14
DB	218 ± 0.02	0.39 ± 0.014	690 ± 18	530 ± 15
DL	196 ± 0.02	0.32 ± 0.007	550 ± 25	270 ± 21

## Data Availability

The data presented in this study are available on request from the corresponding author.
